# An Efficient Topology Discovery Protocol with Node ID Assignment Based on Layered Model for Underwater Acoustic Networks †

**DOI:** 10.3390/s20226601

**Published:** 2020-11-18

**Authors:** Ruiqin Zhao, Yuan Liu, Octavia A. Dobre, Haiyan Wang, Xiaohong Shen

**Affiliations:** 1 Key Laboratory of Ocean Acoustics and Sensing, School of Marine Science and Technology, Northwestern Polytechnical University, Xi’an 710072, China; rqzhao@nwpu.edu.cn (R.Z.); yuan_l@mail.nwpu.edu.cn (Y.L.); xhshen@nwpu.edu.cn (X.S.); 2 Faculty of Engineering and Applied Science, Memorial University, St. John’s, NL A1B 3X5, Canada; odobre@mun.ca; 3 School of Electronic Information and Artificial Intelligence, Shaanxi University of Science and Technology, Xi’an 710021, China

**Keywords:** network topology discovery, ID assignment, underwater acoustic networks

## Abstract

Underwater acoustic networks are widely used in survey missions and environmental monitoring. When an underwater acoustic network (UAN) is deployed in a marine region or two UANs merge, each node hardly knows the entire network and may not have a unique node ID. Therefore, a network topology discovery protocol that can complete node discovery, link discovery, and node ID assignment are necessary and important. Considering the limited node energy and long propagation delay in UANs, it is challenging to obtain the network topology with reduced overheads and a short delay in this initial network state. In this paper, an efficient topology discovery protocol (ETDP) is proposed to achieve adaptive node ID assignment and topology discovery simultaneously. To avoiding packet collision in this initial network state, ETDP controls the transmission of topology discovery (TD) packets, based on a local timer, and divides the network into different layers to make nodes transmit TD packets orderly. Exploiting the received TD packets, each node could obtain the network topology and assign its node ID independently. Simulation results show that ETDP completes network topology discovery for all nodes in the network with significantly reduced energy consumption and short delay; meanwhile, it assigns the shortest unique IDs to all nodes with reduced overheads.

## 1. Introduction

Underwater acoustic networks (UANs) have become increasingly important, with the advancements in underwater communication technologies and their applications in environmental monitoring, exploration of the oceans, and military missions [1,2,3]. UANs, in general, consist of various sensors and vehicles deployed underwater that are connected via acoustic links to perform collaborative tasks [4]. Due to their flexible deployment and huge potential in marine engineering and industry, the study on UANs has become a hot issue.

The network topology discovery is the first step after a UAN is deployed in a marine region because network nodes do not know who are their neighbors or who can communicate with them. Accordingly, they cannot perform specific tasks [5,6,7]. Generally, node ID assignment and network topology discovery are two key tasks for the network establishment, especially in an underwater environment where node position is difficult to obtain [8,9]. Due to the node drifting caused by ocean current and some uncertainty factors during the node deployment, it is difficult to obtain the network topology at each node in advance. Therefore, the network topology discovery procedure is essential for the establishment of underwater acoustic networks [10], being the base for medium access control, routing strategies, topology control algorithms, and other network protocols [11,12,13,14,15].

Generally, the acoustic sensor nodes are networked in a multi-hop style, where data are relayed hop-by-hop [16]. A UAN consists of nodes [17] that communicate with each other through acoustic links, where no centralized infrastructure is generally provided. As the underwater acoustic channel is shared, multiple neighboring nodes sending messages simultaneously would cause collisions and disrupt the message transmission. Thus, nodes have to coordinate their activities in a distributed fashion [18], to ensure a low collision probability during the UAN topology discovery.

However, the unique features of the underwater acoustic network bring the following key challenges to the topology discovery: (1) The delay caused in topology discovery is required to be as small as possible to ensure a fast and timely UAN establishment [19]. However, the large propagation delay of the acoustic channel (the propagating speed of the acoustic signal is approximately 1500 m/s) imposes difficulties to complete the topology discovery with a short delay [20]; (2) The network nodes are placed underwater and they are powered by the battery they carry. Network performance will be affected if the node energy is exhausted and the void hole occurs [21]. To achieve the neighbor discovery in the initial network state, each node needs to send topology discovery (TD) packets several times or increase the transmission power to enhance the probability of being discovered; this makes it challenging to complete the network topology discovery in an energy efficient way [22]; (3) In addition, as the MAC protocol cannot work well in the initial network stage, this unstructured state of the network yields a high probability of packet collisions. An ideal topology discovery protocol should suppress the collision of TD packets effectively. However, as nodes do not have enough neighbor information in the initial network state, i.e., the neighbors’ IDs or their number, it is challenging to achieve the collision suppression during the topology discovery [23]; (4) Typically, unique IDs could be assigned to nodes before the network deployment for a fixed scale UAN. However, for self-organized networks with varying node numbers and multiple networks merging, it is difficult to set a unique ID to each node before the network deployment; a dynamic node ID assignment mechanism is required in such a case to complete the topology discovery [24].

In this paper, we investigate a network topology discovery protocol for UANs to address the aforementioned challenges. The main contributions of this paper are four-fold. First, an efficient topology discovery protocol (ETDP) is proposed to achieve adaptive network node ID assignment and topology discovery simultaneously. Without prior information about the network scale or node IDs, ETDP obtains the entire network topology and short unique node ID for each node with a significantly reduced energy consumption and a short delay. Second, the dynamic topology discovery protocol for UANs is established based on a layered network model, which controls the transmission of TD packets through a random timer design; thus, it significantly suppresses collisions in the initial network state. Exploiting the received TD packets from neighbors, each node obtains the network topology and assigns its node ID independently. Third, a distributed adaptive node ID allocation mechanism for UANs with unknown node numbers is proposed, which allocates the shortest unique node IDs to all nodes with reduced overheads. To the best of the authors’ knowledge, the proposed ETDP is the first protocol that efficiently supports heterogeneous network merging for UANs with different topologies and sizes. Fourth, the OPNET model for underwater acoustic network topology discovery is constructed, and the effectiveness of the proposed protocol is verified through simulations.

The rest of this paper is organized as follows. Section 2 explains relevant research results on node discovery protocols for UANs. Section 3 presents the system model of the network discovery. Section 4 introduces the proposed ETDP protocol and illustrates it through an example. Section 5 theoretically analyzes and calculates the delay and energy consumption of the proposed protocol. A distributed ID assignment and topology discovery protocol for UANs (DIVE) is considered as a benchmark method, and its performance is analyzed. Section 6 outlines the results of the simulation experiments, and Section 7 concludes the paper.

## 2. Related Works

**Figure 1:**
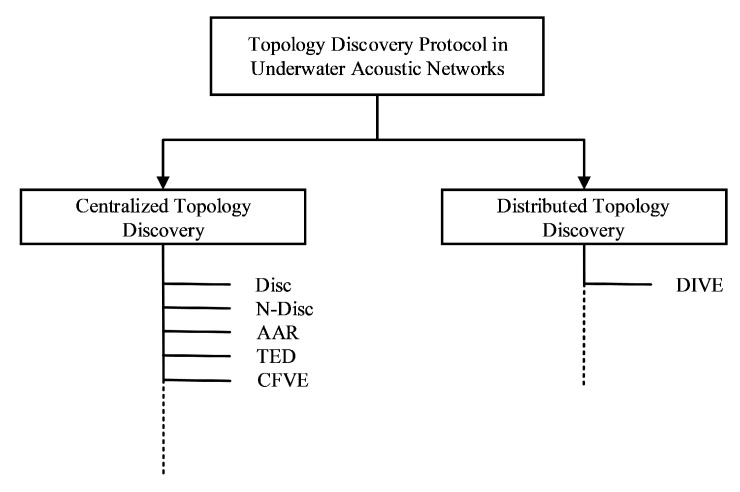
Taxonomy of the existing topology discovery protocols in underwater acoustic networks. Note that Disc [30] is a discovery process for initializing underwater acoustic network (UAN); N-Disc [31] is a node discovery protocol for ad hoc UAN; automatic node ID assignment and resolution protocol for UANs (AAR) [34] is an address assignment and resolution protocol for UANs; topology-efficient discovery algorithm (TED) [32] is topology-efficient discovery algorithm for UANs; CFVE [33] is a collision-free topology discovery protocol; and DIVE [35] is a distributed ID assignment and topology discovery protocol for UANs.

The problem of topology discovery for wireless sensor networks (WSNs) has been extensively studied [25,26,27,28]. However, the topology discovery for UANs is more complicated due to the harsh underwater acoustic channel. This section discusses existing topology discovery protocols in UANs. A taxonomy of existing topology discovery protocols in UANs is presented in Figure 1. According to the network model, node discovery protocol can divided into centralized discovery and distributed discovery.

In the centralized network architecture, there are central nodes that start and control the operation of the protocol. In [29], a primary seed node (node with known co-ordinates) is used to determine secondary and tertiary seed nodes. Co-ordinates of the first three seed nodes are used to build up the relative co-ordinate system through the triangulation method. In [30], the discovery process (Disc) is centrally controlled by a node designated as master node. Each ordinary node that discovers its neighbors sends this information back to the master node. Thus, the master node acquires the newest topology quickly. However, this also results in increased energy consumption. Furthermore, in [31], leader nodes are proposed to control the discovery process (N-Disc). Considering the limited energy of UANs, the transmit power is classified in several levels according to the propagation distance. Although the protocol can reduce the energy consumption after the network topology is obtained, the energy consumption and discovery delay of the protocol are not satisfying. The reason is that nodes need to send packets from low to high power levels several times to determine the minimum transmitting power level for each link. The topology-efficient discovery algorithm (TED) in [32] allows nodes to share time slots while controlling the number of possible collisions such that the delay of the topology discovery process can be reduced. In [33], a collision-free topology discovery protocol is proposed, where each node transmits its discovery packets at a unique time. This approach can also reduce the energy consumption of the network topology discovery.

Opposite to the centralized network architecture, there are no central nodes that start and control the operation of the protocol in a distributed configuration. A design of an automatic node ID assignment and resolution protocol for UANs (AAR) is proposed in [34]. In [35], a distributed ID assignment and topology discovery protocol for UANs is proposed. While assigning the node IDs, additional information is shared to discover the other nodes in the network. The protocol is composed of two main procedures that run simultaneously. The first one is to share the information required to assign the node IDs and to discover the network topology. The second procedure is required to verify that node IDs are unique in the network. Due to the distributed discovery approach, the nodes start working at the same time, resulting in a smaller discovery delay for the distributed protocols than for the centralized protocols. In [36], a neighbor discovery mechanism is presented based on directional transmission and reception.

Table 1 provides a comparative summary of the previously discussed protocols. TED and CFVE protocols require time synchronization because they employ time division multiple address (TDMA). Most of the discovery protocols require knowledge of the number of network nodes. The criteria to measure the protocol performance includes link connections, energy consumption, convergence time or delay, the number of discovered nodes, and transmitted packets. The CFVE and DIVE protocols can adapt to node mobile scenario, while others require settled nodes. Besides, the existing discovery protocols require knowledge of the node ID in advance except for the DIVE protocol. Considering these aspects, our protocol will be compared with the DIVE protocol.

## 3. System Model

A UAN can be abstracted as a graph G(V,E), where *V* is the set of all nodes in the network and *E* consists of all links of the network. Some definitions are as follows:

1. ∀i,j∈V is the Euclidean distance between node *i* and node *j*.
2. $P_{t}$ and $P_{r}$ are the transmit and receive power of all nodes in the network, respectively. All nodes have the same transmit power $P_{t}$.
3. $d(P_{t})$ represents the transmission range of each node with transmit power $P_{t}$.
4. Neighbor(i) consists of node *i*’s neighbors that could receive TD packets directly from node *i*.

The UAN spans multiple hops, which can be presented with a tree-based topology structure. Node *T* is the root node, neighbors of the root node belong to layer l, layer 1 nodes’ children belong to layer 2, and so on. Thus, the entire network is layered into the root node, layer 1, layer 2, …, layer $L_{i}$, …, and layer *L*. $L_{i}$ is the layer of node *i*.

The following definitions are additionally used:

1. Root node: The root node triggers the beginning of the ETDP.
2. Father node and child node: When node *i* firstly receives a TD packet from node *j* that is in layer $L_{i}-1$, node *i* sets node *j* as its father node. In the same way, when node *j* receives a TD packet from a layer $L_{i}$ node, whose father node is node *j*, node *j* sets it as one of its child nodes. In this network model, each node has one father node; however, it may have several child nodes. Besides, each node can be a father node and a child node at the same time, except for the root node and leaf nodes.
3. Leaf node: Nodes that do not have any child nodes are called leaf nodes.
4. Descendant node: Node *i*’s descendant nodes include all its child nodes and their offspring nodes, which generally have a larger layer number than node *i*.

To efficiently complete topological discovery and node ID allocation based on a tree structure, all nodes have a unique parent node except for the root node. $L_{i}$ is the layer of node *i*. $D_{i}$ is the number of descendant nodes of node *i*, $C_{i}$ is the number of child nodes of node *i*.

**Figure 2:**
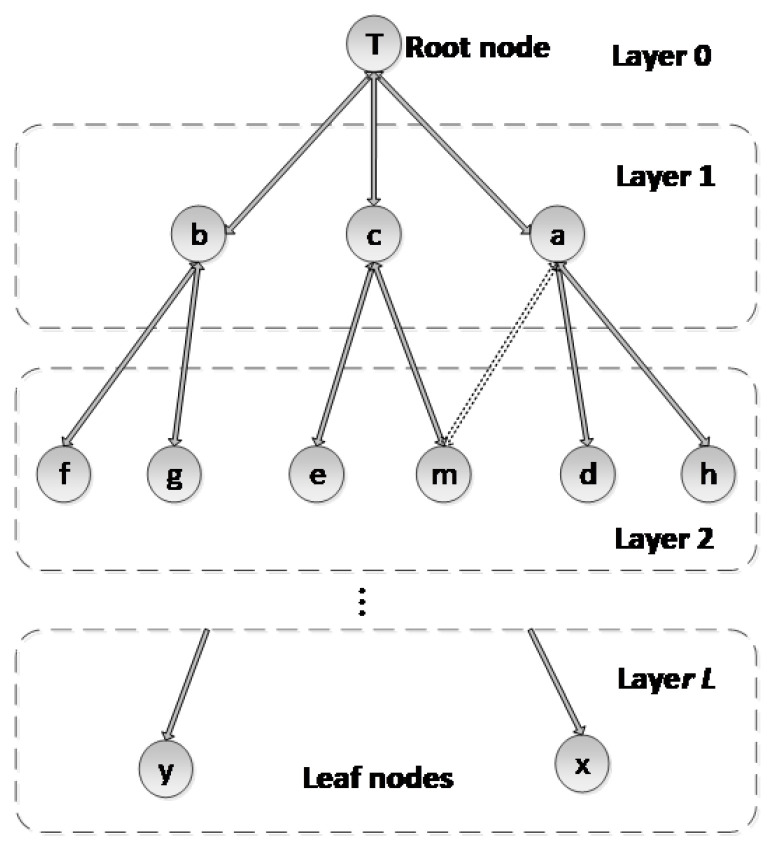
The network model.

For example, as shown in Figure 2, *T* is the root node and its layer is 0. Nodes within its transmission range (e.g., nodes *a*, *b* and *c*) are its neighbor nodes, and their layer is 1. As nodes *a*, *b* and *c* have received the TD packet from the root node for the first time, the root node is the father node of *a*, *b* and *c*. In the same way, as the root node can receive the packets from *a*, *b* and *c*, these are neighbors and child nodes of the root node. Accordingly, the ordered pair of nodes (rootnode|a,b,c) shows that *a*, *b* and *c* are child nodes of the root node and the root node is the father node of nodes *a*, *b* and *c*. Nodes *x* and *y* are leaf nodes because they do not have child nodes. Based on the above definition for the descendant node, all nodes except for the root node are descendant nodes of node *T*.

## 4. Proposed ETDP Protocol

Our goal is to achieve topology discovery and adaptive node ID assignment simultaneously with reduced energy consumption and delay.

The following assumptions are made:

1. The number of nodes in the network and the states of connectivity are unknown.
2. The node IDs have not been assigned.
3. One node is set as the root node, which triggers the topology discovery procedure.
4. Nodes communicate in half-duplex mode.

In the initial stage of the network, nodes are unstructured and do not know the information about the surrounding nodes and links. To reduce the energy consumption and delay caused by the unknown network topology, the ETDP protocol establishes the network layered structure through the transmission of TD packets based on a local timer, where nodes are classified into different layers and defined as the root node, father nodes, child nodes, leaf nodes, and descendant nodes, as mentioned in Section 3. Hence, the dynamic topology discovery protocol for UANs could be established based on a layered network model, which controls the transmission of TD packets through a random timer design, and thus, significantly suppresses collisions in the initial network state. Exploiting the received TD packets from neighbors, each node obtains the network topology and assigns its node ID independently.

Two timers are defined for node *i*: $Tx\_Timer_{i}$ and $Wait\_Timer_{i}$. The former is employed to reduce the packet collision probability and the latter is used to collect the descendant topology of the node. The two timers are defined as

(1) $$Tx\_Timer_{i}=K_{i}/100,$$

(2) $$Wait\_Timer_{i}=\left(L-L_{i}+1\right)\cdot \left(2T_{p}+2T_{c}+Tx\_Timer_{max}\right),$$

where $K_{i}$ is a random value generated by node *i*, $T_{c}$ is the link propagation delay of the TD packet, $T_{p}$ is the transmission delay for the HELLO packet, and $Tx\_Timer_{max}$ is the maximum Tx_Timer.

**Figure 3:**
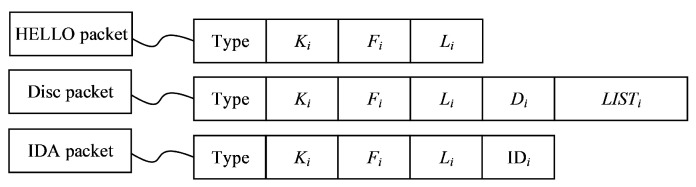
TD packet formats in the ETDP protocol.

The TD packet includes three types of packets, i.e., HELLO, Disc (discovery), and IDA (ID assignment) packets, which are shown in Figure 3. Here, Type is the TD packet type, $F_{i}$ is the $K_{i}$ value of the node *i*’s father node, $ID_{s}$ is the ID of the sending node, and $LIST_{i}$ shows the partial topology that includes all descendant nodes and neighbor nodes of node *i*.

The detailed algorithm is shown in Algorithm 1. Accordingly, the ETDP protocol consists of three stages: HELLO packet transmission, disc packet transmission, and IDA packet transmission. Firstly, the network is layered and neighbor discovery is achieved through the HELLO packet transmission stage. Then, the entire network topology discovery could be completed at the end of the disc packet transmission stage. Finally, each node computes its unique ID based on the topology information and sends the IDA packet in the last stage.
**Algorithm 1** Topology Discovery Algorithm.**Inputs:**rootnodeT, $K_{T}=0$, $L_{T}=0$.**Outputs:** topology discovery process.1:**HELLO:** node *T* sends HELLO and starts $Wait\_Timer_{T}$;2:   **while** (ordinary node *i* receives HELLO)3:   {makes sure its fathernode and Li; generates Ki; updates its HELLO; starts $Tx\_Timer_{i}$;}4:      **while** ($Tx\_Timer_{i}$ expires)5:      {send HELLO, set $Wait\_Timer_{i}$;}6:   **while** (a father node receives HELLO)7:   {updates its childnode;}8:   **while** (completion)9:   { get $L_{i}$, childnode, fathernode, leafnode; break; }10:**Disc: while** (node *i*’s $Wait\_Timer_{i}$ expires)11:      {**if** (has not received any HELLO)12:      **then** set *i* as leafnode; generate disc packet and send it to $F_{i}$;13:            **else** update and send disc packet to $F_{i}$;}14:      **while** (completion)15:      {break; get its descendant topology and $D_{i}$;}16:**IDA: while** ( $Wait\_Timer_{T}$ expires)17:      {set 0 as ID, update information and send IDA packet;}18:      **while** (ordinary node *i* receives IDA packet)19:      {compute ID, update information and start $Tx\_Timer_{i}$;20:            **while** ($Tx\_Timer_{i}$ expires)21:            {send IDA packet;}}22:      **while** (completion)23:      { get node $ID_{i}$ and network topology; break;}24:end;

### 4.1. HELLO Packet Transmission Stage

The root node *T* initiates the topology discovery process by sending a HELLO packet according to the network TD packet formats shown in Figure 3, with $K_{T}=0$ and $L_{T}=0$. Then, it starts the $Wait\_Timer_{T}$. Each node that has received a HELLO packet for the first time from the root node would set the root node as its father and set its layer as 1. Then, these nodes generate a random value $K_{i}$ as well as their HELLO packet and start the $Tx\_Timer_{i}$ based on Equation (1). After the timeout of $Tx\_Timer_{i}$, they send their HELLO packets to their neighbors and start the $Wait\_Timer_{i}$. Similar operations are performed at the following layers. As such, each node sends its HELLO packet and starts the $Wait\_Timer_{i}$ until reaching the nodes at layer *L*. Therefore, the HELLO packet transmission stage performs the above operations, starting from the root node up to the leaf node of the network. After all network nodes complete the HELLO packets transmission stage, the network is layered into layer 0, layer 1, …, layer $L_{i}$, …, layer *L* and all leaf nodes are confirmed. Meanwhile, each node has its child node and father node, except for the leaf nodes and the root node, respectively.

At this stage, the $Tx\_Timer_{i}$ is associated with a random value to enable nodes at the same layer to send packets at different times, which reduces the possibility of packet collisions.

### 4.2. Disc Packet Transmission Stage

ETDP defines the node that has not received any HELLO packets after the $Wait\_Timer_{i}$ timeout as a leaf node. When the $Wait\_Timer_{i}$ expires, the leaf nodes generate a disc packet and send it to their father node. The node that receives its child node disc packet stores information and updates its disc packet. The father node collects all disc packets from its children during the $Wait\_Timer_{i}$, and updates and sends a disc packet to the upper-layer node when the $Wait\_Timer_{i}$ expires. After all nodes have sent disc packets, the network topology information is gathered at the root node.

At this stage, the $Wait\_Timer_{i}$ ensures that each node receives packets from all its neighbors. After this stage is completed, each node in the network has more information of the network topology than at the previous stage. Each node *i* knows all its descendant topology and the number of its descendant nodes $D_{i}$.

### 4.3. IDA Packet Transmission Stage

The root node sets 0 as its node ID. Then, it transmits the obtained network topology as a single aggregated packet IDA to layer 1 nodes. After receiving an IDA packet from the father node, node *i* starts the $Tx\_Timer_{i}$ based on Equation (1) and checks whether the $K_{i}$ value of node *i* is included in this packet. If so, it calculates its node ID according to Equations (3)–(5). Then, it generates its IDA packet and sends it after the $Tx\_Timer_{i}$ expires. Otherwise, node *i* does nothing.

Each node *i* assign its $ID_{i}$ according to

(3) $$K_{i}=R,$$

(4) $$ID_{i}=\sum_{R=0}^{R-1}D_{Li}^{R-1}+ID_{f}+R,\;C_{i}>1,$$

(5) $$ID_{i}=ID_{f}+1,\;C_{i}=1,$$

where *R* is the order of nodes’ $K_{i}$ values in the same layer $L_{i}$, $ID_{f}$ denotes the father ID of node *i*, and $D_{Li}^{R}$ is the number of descendants of node *i* whose $K_{i}$ order is *R* at layer $L_{i}$. We define $C_{i}$ as the number of node *i*’s children. The node obtains the unknown parameters in the Equations (3)–(5) during the shared TD packets and calculates its ID.

### 4.4. Illustration of EDTP

**Figure 4:**
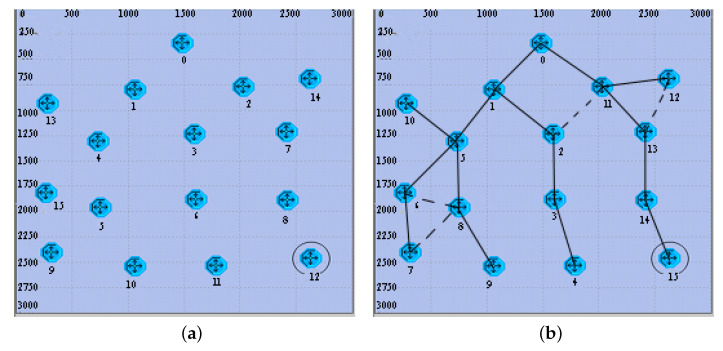
The network connectivity achieved after ETDP procedure. (**a**) A random network with no structure; (**b**) Topology acquired after the ETDP is completed.

We now illustrate the steps of the ETDP procedure with a 12 nodes network. Figure 4a shows a random network that has no structure. In this network, node 0 is the root node and node IDs are unknown. After all nodes are randomly deployed, the overall structure and connectivity of the network are unknown. Compared with the node deployment of the actual network, Figure 4b shows the topology acquired and ID assignment after the ETDP procedure is completed.

Table 2 shows the detail of ETDP process. In this example, in the HELLO packet transmission stage, node 0, which sets its layer to 0, sends HELLO and starts the $Wait\_Timer_{0}$. After broadcasting the HELLO packet, node 0 neighbors (nodes 1 and 2) receive node 0’s HELLO. They set their layer to 1 and set node 0 as their father node. After nodes 1 and 2 generate $K_{1}=20$ and $K_{2}=30$, they start $Tx\_Timer_{1}=0.2$ s and $Tx\_Timer_{2}=0.30$ s, respectively. When the timers expire, they transmit their HELLO packet and start $Wait\_Timer_{1}$ and $Wait\_Timer_{2}$, separately. Then, node 0 receives the HELLO packets from nodes 1 and 2 and stores its two children nodes. Similarly, when nodes 3 and 4 receive a HELLO packet from node 1, they set their layer to 2 and store node 1 as their father. In this example, nodes 3, 7 and 14 can receive the HELLO packet from node 2. Because node 3 already has a father node, node 3 identifies node 2 as its neighbor. Only nodes 7 and 14 set node 2 as their father. After nodes 3 and 4 generate $K_{3}=78$ and $K_{4}=96$ at random, they start the $Tx\_Timer_{i}$. When this expires, they transmit the HELLO packet and start the $Wait\_Timer_{i}$. As time goes on, every node sends its HELLO packet. Table 2 provides a detailed summary of the system states. ETDP uses $T\_ID_{i}<K_{i},\;F_{i},\;L_{i}>$ as the temporary ID of different nodes (e.g., $T\_ID_{1}<20,\;0,\;1>$ represents node 1). Therefore, node 0 child nodes are nodes $T\_ID_{1}$ and $T\_ID_{2}$. Nodes 14, 7, 13, 11, 9, 10, 12 are leaf nodes, as the number of their children is zero. So, node 12 sets its descendant to 0. When its $Wait\_Timer_{12}$ expires, node 12 sends the disc packet to the father node 8. After node 8 receives the disc packet from its child node 12, it calculates its $D_{8}=1$. When its $Wait\_Timer_{8}$ expires, node 8 sends the disc packet to its father node 7. After node 7 receives the disc packets from its child node 8, it computes it. Then, when its $Wait\_Timer_{7}$ expires, node 7 sends the disc to its father node 2. Node 2 collects the disc packets from its children nodes 7 and 14 and calculates its Similarly, when its $Wait\_Timer_{2}$ expires, node 2 sends the disc packets to its father node 0. Finally, when $Wait\_Timer_{0}$ expires, node 0 collects the disc packets of its children nodes 1 and 2. Then, it ends the disc transmission process and the entire network information is gathered at node 0.

After $Wait\_Timer_{0}$ expires, node 0 sends the IDA packet for ID assignment and topology notification. The ID of node 0 is set to 0. In our example, node 0 has two child nodes 1 and 2 in Figure 4a, which compute their IDs according to Equations (3)–(5) when they received IDA packets. Therefore,

(6) $$\left\{\begin{array}{cc} \; & node\;1\to K_{1}=20,\;R=1,\\ \; & node\;2\to K_{2}=30,\;R=2, \end{array}\right.$$

(7) $$\left\{\begin{array}{cc} \; & ID_{1}=\sum_{R=1}^{0}D_{1}^{0}+ID_{f}+R=0+0+1=1,\\ \; & ID_{2}=\sum_{R=1}^{2}D_{1}^{2-1}+ID_{f}+R=9+0+2=11. \end{array}\right.$$

Analogously, node $ID_{2}=11$ has two child nodes 7 and 14 in Figure 4a. After they received the IDA packet from node 2, they computed their IDs as $ID_{7}=13$ and $ID_{14}=12$, respectively. After the TD packets transmission and ID assignment, all nodes in the network are assigned a unique address and obtain network topology information.

## 5. Theoretical Analysis

The performance of the proposed ETDP protocol is evaluated through analysis, when considering single-hop and multi-hop UANs. Two metrics are investigated to assess the effectiveness and overhead of the ETDP protocol, as follows:

- Topology discovery communication traffic: The total communication traffic consumed by network nodes to complete the network topology discovery.
- Topology discovery duration: The interval from the beginning of the network topology discovery until all nodes in the network obtain the entire topology information.

Meanwhile, the compared protocol DIVE is analyzed, including its communication traffic and discovery duration. Then, it is theoretically proven that the ETDP algorithm discovery communication traffic and discovery duration are better than for the existing DIVE protocol.

### 5.1. Topology Discovery Communication Traffic

For the network topology discovery phase, overhead is an indicator of the protocol performance, which has a linear relationship with the energy expenditure. Let us denote by *N* the total number of nodes and by $N^{i}$ the number of neighbors of node *i* in the network. In the HELLO packet transmission stage, the communication traffic is

(8) $$bit_{1}=\sum_{i=0}^{N}\left(N+N\cdot N^{i}\right)\cdot \left(2+log_{2}K_{i}+log_{2}F_{i}+log_{2}L_{i}\right),$$

while in the disc packet transmission stage, this is

(9) $$bit_{2}=\left(2N-1\right)\cdot \sum_{i=0}^{N}\left[2+\left(N+1\right)\cdot \left(log_{2}K_{i}+log_{2}F_{i}+log_{2}L_{i}\right)+log_{2}N\right],$$

and finally, in the IDA packet transmission stage, it is

(10) $$bit_{1}=\sum_{i=0}^{N}\left(N+N\cdot N^{i}\right)\cdot \left(2+log_{2}K_{i}+log_{2}F_{i}+log_{2}L_{i}+log_{2}N\right),$$

The total communication traffic required to complete the node ID assignment and topology discovery process is $\sum_{i=1}^{3}bit_{i}$.

The DIVE protocol is composed of two procedures of the network topology discovery and node ID assignment. In its first procedure, HELLO packets are shared to discover information and assign the node IDs. Table 3 illustrates the HELLO packet length of the DIVE protocol. The discovery HELLO packet format of the benchmark protocol DIVE is

$$HELLO_{x}=\left\{K_{x},R_{x},isMobile_{x},C_{x},LIST_{x}\right\},$$

where $K_{x}$ is the key value generated by node *x*; $R_{x}$ is an additional random integer value. $isMobile_{x}$ is a flag set to 1 if node *x* is mobile, and to 0 otherwise; $C_{x}$ is a counter to track how many HELLO packets have been transmitted by *x* so far; $LIST_{x}$ is the list of contacts stored by node *x*. Therefore, the communication traffic required by the DIVE protocol to complete the network topology discovery process is

(11) $$c_{t}=\left[31+10N+\left(N-1\right)\left(30+log_{2}N\right)\right]\cdot N_{p},$$

where $N_{p}$ is the total number of sent packets in the network.

The paper calculates the communication traffic of the two protocols during topology discovery process. As such, the theoretical energy consumption is derived in Section 6.

### 5.2. Topology Discovery Delay

The goal of topology discovery is that each node of the network obtains the entire network topology and a unique shortest ID.

Topology discovery duration comes from three stages: HELLO packet transmission, disc packet transmission, and IDA packet transmission.

In the HELLO packet transmission stage, the HELLO packet is sent hop-by-hop from the root node until the leaf node. In the disc packet transmission stage, nodes send disc packets hop-by-hop from the leaf node to the root node. In the IDA packet transmission stage, the IDA packet is sent hop-by-hop from the root node until the leaf node. The time required by these three processes is

(12) $$T_{total}=Wait\_Timer_{0}+L\cdot \left(Tx\_Timer_{Li}^{max}+T_{p}+T_{c}\right),$$

where $Tx\_Timer_{Li}^{max}$ is the maximum $Tx\_Timer_{Li}$ at layer of $L_{i}$.

Regarding the discovery duration of the DIVE protocol, it is composed of two main procedures that run simultaneously. The first one is to share the information required to assign the node IDs and to discover the network topology. The second procedure is required to verify that node IDs are globally unique in the network.

(13) $$\left\{\begin{array}{cc} \; & \left(2+N_{c}\right)\cdot \left(K\mu_{i}+\frac{C_{t}}{R}+\frac{L}{c}+\tau_{G}\right)\leq T_{i}\\ \; & \leq \left(N_{max}^{i}+1+N_{c}\right)\cdot \left(K\mu_{i}+\frac{C_{t}}{R}+\frac{L}{c}+\tau_{G}\right), \end{array}\right.$$

where $C_{t}$ is the size of the HELLO packet, *R* is a nominal transmission rate, *L* is propagation distance, *c* is the speed of sound, and $\tau_{G}$ is the guard interval. DIVE is a distributed protocol in which each node starts discovery at same time. Thus, node *i* requires $T_{i}$ to complete the discovery procedure shown as Equation (13), where $N_{max}^{i}$ is the maximum $N^{i}$, $\mu_{i}$ is a coefficient, and $N_{c}$ is the number of collisions at node *i*. For the DIVE protocol, the discovery duration depends on the maximum $T_{i}$ between the network nodes.

## 6. Simulation Evaluation

The performance of the proposed ETDP protocol is evaluated via OPNET simulation where the pipe stage of underwater acoustic channel is adopted. In our simulation evaluation, nodes are randomly distributed in an area of 3000 m × 3000 m, where the node communication distance is 700 m and data rate is 7500 bps. The size of network is constant with the number of nodes increases. The number of nodes is varying between 4 and 200. When the maximum number of nodes is considered, the maximum number of layers is 5. The transmission, reception, and idle powers are set to 8 W, 1.3 W, and 0.285 W, respectively, as shown in Table 4. To investigate the influence of the harsh underwater acoustic channel, packet error ratio (PER) of acoustic links is considered in the simulations.

**Figure 5:**
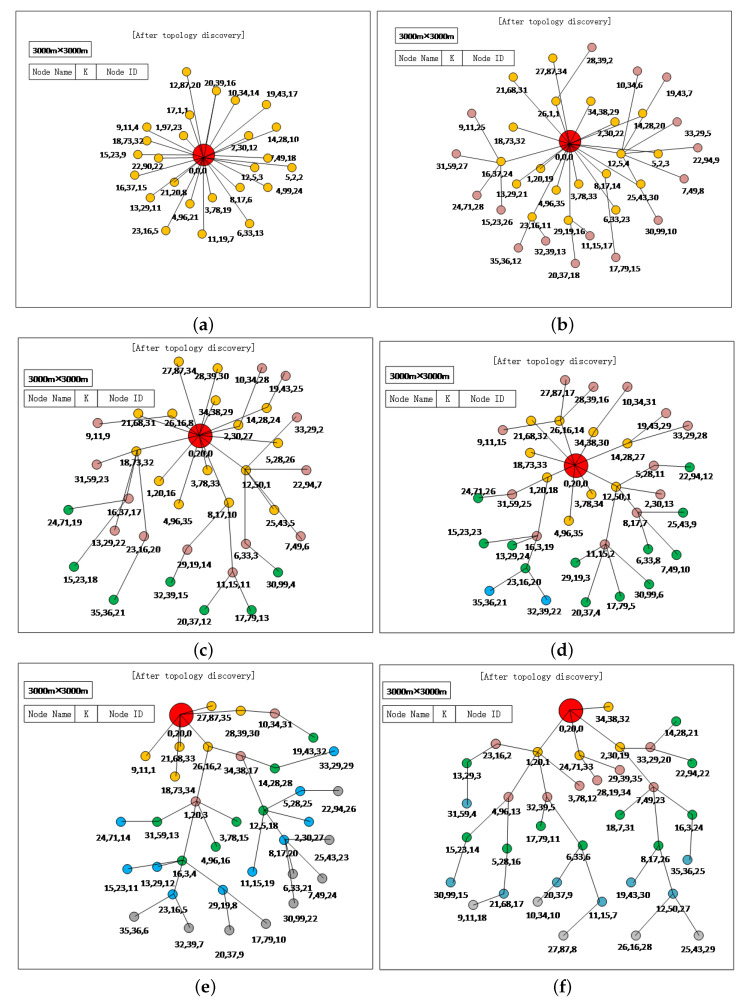
Acquired various network topology by the proposed ETDP protocol considering different node deployment: (**a**) Network layer is 1; (**b**) Network layer is 2; (**c**) Network layer is 3; (**d**) Network layer is 4; (**e**,**f**) Network layer is 5.

We consider different node deployments to analyze the performance of ETDP. The network topology obtained through the proposed ETDP protocol is displayed in OPNET ODB (OPNET Simulation Debugger) window. Figure 5 shows how the connections between the nodes change according to the different number of network layers. Initially, we deploy a network with the maximum layer 1, then the nodes organize a star-topological network by EDTP successfully (Figure 5a). Secondly, we keep the number of nodes unchanged and increase the maximum hop count to 2 (Figure 5b), 3 (Figure 5c), 4 (Figure 5d) and 5 (Figure 5e,f), respectively. The simulation results show that the network topology can be fully discovered under different node deployment scenarios. We use different colors to represent different layers of nodes. For the convenience of understanding the discovery process, we did not draw the neighbor information of each node; only the nodes with the parent–child relationship were drawn in the Figure 5.

The results show that ETDP can obtain entire network topology with different network size. However, it only obtains neighbor information, number of hops and total number of nodes in DIVE. The discovered topology assumes a star state when the network layer size is small. As the maximum number of layers of the network increases, the discovered network topology appears as a tree. At the same time, for different network topologies, all nodes in the network share TD packets and generate their random values $K_{i}$, i=1,…,N. Finally, they complete topology obtaining and the random value $K_{i}$ helps network nodes to successfully assign the unique and shortest ID.

**Figure 6:**
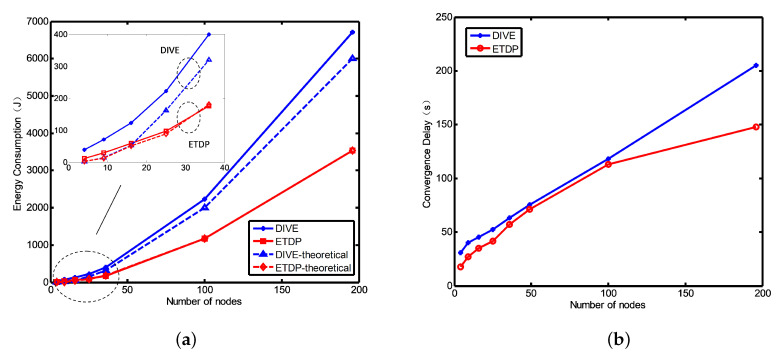
The performance comparison between the proposed ETDP and DIVE protocols: when PER = 0, (**a**) Energy consumption for network topology discovery; (**b**) Network topology discovery convergence duration.

We consider the performance of ETDP in terms of energy consumption and convergence delay, and compare it with that of the DIVE algorithm under the same simulation environment and parameters. Figure 6a,b show the total energy consumption and convergence delay versus the number of nodes; these increase as the number of nodes in the network increases. As shown in the Figure 6a, the energy consumption of the proposed ETDP protocol is significantly lower than that of DIVE as the number of nodes increases. ETDP effectively decreases the number of transmission TD packets by using the relationship between the parent and child nodes. After the HELLO packet transmission stage, a (fathernode|childnodes) relationship is established between nodes, which can partly reduce the unordered collision of TD packets in the initial stage of the network. On the other hand, the protocol has two timers to ensure that the node receives enough TD packets before replying, which reduces the packet transmissions in the network. Thus, the overall optimization of the discovery strategy effectively reduces the energy consumption. As the value of $K_{i}$, $L_{i}$ and $F_{i}$ in ETDP are randomly generated values, we directly computed the mean for these values in the theoretical analysis. When the number of nodes in the network increases gradually, the mean value is closer to the actual value in ETDP. Whereas, DIVE generates more random packet collisions as the number of nodes increases. The packet retransmission caused by the collisions is not considered in the theoretical analysis in DIVE protocol, resulting in a deviation between the theoretical value and the actual value in DIVE.

After the theoretical analysis of duration, the topology discovery duration of the proposed ETDP protocol is related to the number of layers in the network. The increase of network node number may cause the increased network layer, which extends the convergence delay of ETDP, as shown in Figure 6b. Besides, ETDP uses a relatively less random value and smaller packet size, which reduced the delay to some extent compared with DIVE. In summary, the optimized discovery cycle reduces the number of transmitted packets. Thus, compared to the benchmark method, the proposed protocol obtains the network topology while greatly reducing the time and energy overhead.

**Figure 7:**
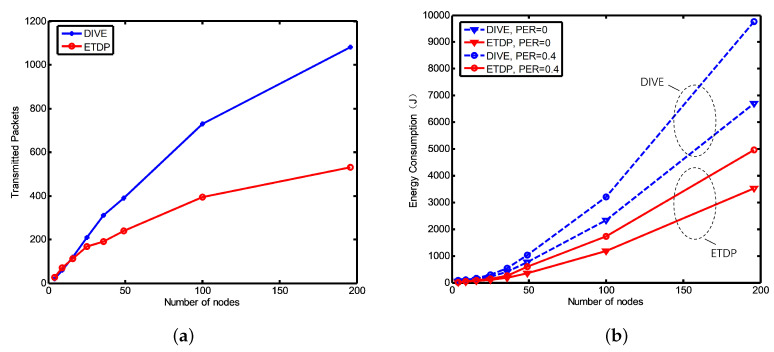
(**a**) The number of transmitted TD packets, PER = 0; (**b**) The network topology discovery energy consumption versus packet error probability.

The number of transmitted TD packets is shown in Figure 7a versus the number of nodes. Results show that when the number of network nodes is small, the number of packets in the ETDP three-stage discovery strategy is slightly larger than for DIVE, for which the number of packet collisions and packet exchanges is lower. On the other hand, when the number of nodes increases, the proposed ETDP protocol outperforms DIVE. Due to the layered mechanism, an increase in the number of nodes does not cause a significant increase in the *K* value of ETDP. However, the packets sending strategy of DIVE causes notable enlargement of packet collision probability as the number of nodes increases.

To investigate the influence of the harsh underwater acoustic channel, the energy consumption of the two protocols is compared under different packet error ratio (PER), as shown in Figure 7b. With the increasing node number and PER, the energy consumption of two protocols increases. It can be seen that the energy consumption of DIVE is higher than that of ETDP at the same network size and PER, as ETDP has a lower number of packages sent and reduced package sizes. Moreover, results show that the increase in the number of nodes has a relatively larger effect than PER on the energy consumption of the two protocols. Furthermore, the energy consumption of DIVE increases faster than that of ETDP, as the mechanism of distributed flooding is more sensitive to the increase in the number of nodes in DIVE.

## 7. Conclusions

In this paper, the problem of obtaining topology information in UANs has been studied, which is required to determine destination nodes, perform routing, and schedule transmissions efficiently. The ETDP protocol has been proposed as a solution to the network topology discovery and node ID assignment. Through TD packet transmission, a layered structure has been built to avoid packets collisions. Moreover, two timers have been proposed to collect packets. Besides, a node IDs assignment has been introduced, based on the layered structure and collected topology information. It has been revealed that ETDP obtains accurate topology information while greatly reducing the overhead and energy consumption. Simulation results have shown that the proposed ETDP protocol completes the ID assignment for all nodes in the network using the collected topology information. Considering the energy constraint of UAN nodes, the proposed ETDP protocol is a suitable network topology discovery mechanism for UANs. Our future work will concentrate on merging the processes for new nodes joining the network and integrating topology discovery with node localizations with reduced overheads.

## Figures and Tables

**Table 1:** Comparison of the Existing Topology Discovery Protocols for UANS.

Protocols	Synchronous Required	Network Node Number Required	Criteria	Mobility Support	IDs-Aware
Disc [29]	No	Yes	Link connections	No	Yes
N-Disc [30]	No	Yes	Energy, delay, no. of node discovery	No	Yes
TED [31]	Yes	Yes	No. of packet collisions, delay, no. of NFNPs	No	Yes
CFVE [32]	Yes	Yes	Energy, delay, transmitted packets	Yes	Yes
DIVE [34]	No	No	Energy, delay, transmitted packets	Yes	No

**Table 2:** The proposed protocol operation and state information for each node.

No.	$K_{i}$	$F_{i}$	$L_{i}$	$K_{i}$	$F_{i}$	$L_{i}$	Child Node	Neighbor(*i*)	$D_{i}$	$K_{i}$	$F_{i}$	$L_{i}$	${\mathrm{ID}}_{i}$
0	0	—	0	0	—	0	(1, 2)	—	15	0	—	0	0
1	20	0	1	20	0	1	(3, 4)	—	9	20	0	1	1
2	30	0	1	30	0	1	(14, 7)	—	4	30	0	1	11
3	78	20	2	78	20	2	(6)	(2)	2	78	20	2	2
4	96	20	2	96	20	2	(13, 15, 5)	—	5	96	20	2	5
14	28	30	2	28	30	2	—	(7)	0	28	30	2	12
7	49	30	2	49	30	2	(8)	(14)	2	49	30	2	13
6	33	78	3	33	78	3	(11)	—	1	33	78	3	3
13	29	96	3	29	96	3	—	—	0	29	96	3	10
15	23	96	3	23	96	3	(9)	(5)	1	23	96	3	6
5	28	96	3	28	96	3	(10)	(15, 9)	1	28	96	3	8
8	17	49	3	17	49	3	12	—	1	17	49	3	14
11	15	33	4	15	33	4	—	—	0	15	33	4	4
9	11	11	4	11	11	4	—	(5)	0	11	11	4	7
10	34	28	4	34	28	4	—	—	0	34	28	4	9
12	5	17	4	5	17	4	—	—	0	5	17	4	15

**Table 3:** The HELLO Packet Length of DIVE Protocol.

Content	$K_{x}$	$R_{x}$	${\mathrm{isMobile}}_{x}$	$C_{x}$	${\mathrm{LIST}}_{x}$
**Length (bits)**	20	10	1	10N	$\left(N-1\right)(31+log_{2}\left(N-1\right))$

**Table 4:** Network Simulation Parameter Setting.

Simulation Parameter	Parameter Value
Deployment area	3000 m × 3000 m
Transmit power	8 W
Receive power	1.3 W
Idle power	0.285 W
Node communication distance	700 m
Data rate	7500 bps
$Tx\_Timer_{max}$	1 s
*K*	(1, 100)
Number of nodes	N=4,9,16,25,36,49,100,200
Packet error ratio	PER = 0, 0.4
